# A Bio-Inspired Dopamine Model for Robots with Autonomous Decision-Making

**DOI:** 10.3390/biomimetics9080504

**Published:** 2024-08-21

**Authors:** Marcos Maroto-Gómez, Javier Burguete-Alventosa, Sofía Álvarez-Arias, María Malfaz, Miguel Ángel Salichs

**Affiliations:** Department of Systems Engineering and Automation, University Carlos III of Madrid, Av. de la Universidad, 30, 28911 Leganes, Madrid, Spain; jburguet@pa.uc3m.es (J.B.-A.); sofalvar@pa.uc3m.es (S.Á.-A.); mmalfaz@ing.uc3m.es (M.M.); salichs@ing.uc3m.es (M.Á.S.)

**Keywords:** dopamine model, autonomous behaviour, robotics, bio-inspiration, reinforcement learning, pleasure

## Abstract

Decision-making systems allow artificial agents to adapt their behaviours, depending on the information they perceive from the environment and internal processes. Human beings possess unique decision-making capabilities, adapting to current situations and anticipating future challenges. Autonomous robots with adaptive and anticipatory decision-making emulating humans can bring robots with skills that users can understand more easily. Human decisions highly depend on dopamine, a brain substance that regulates motivation and reward, acknowledging positive and negative situations. Considering recent neuroscience studies about the dopamine role in the human brain and its influence on decision-making and motivated behaviour, this paper proposes a model based on how dopamine drives human motivation and decision-making. The model allows robots to behave autonomously in dynamic environments, learning the best action selection strategy and anticipating future rewards. The results show the model’s performance in five scenarios, emphasising how dopamine levels vary depending on the robot’s situation and stimuli perception. Moreover, we show the model’s integration into the Mini social robot to provide insights into how dopamine levels drive motivated autonomous behaviour regulating biologically inspired internal processes emulated in the robot.

## 1. Introduction

Decision-making systems play an essential role in numerous applications related to economics [1], healthcare [2], and robotics [3]. These systems allow artificial agents to behave autonomously by adapting to changing conditions, anticipating previously experienced situations, and interacting with complex environments [4].

Recently, robotics has enhanced numerous assistive applications, improving the quality of life for many people, and helping them in their daily activities. However, real scenarios are complex and require these systems to understand the environment and produce appropriate behaviour according to the different situations they experience. Solving this challenge requires decision-making systems with powerful capabilities, especially in workspaces with limited workforce [3].

Different reviews [2,3] highlight the positive effects of autonomous social robots in reducing staff involvement in healthcare or education, allowing robots to operate in private environments, and improving the user experience by avoiding continuous supervision. The previous facts reveal that social robots contribute to society in many areas where exhibiting social skills facilitates human–robot interaction. Therefore, emulating human behaviour and complying with social norms to approach users and meet their expectations is essential if robots aim to coexist with people.

The design of biologically inspired autonomous robots for human–robot interaction combines neuroscience with robotics [5] to enhance robot capabilities. Neuroscience is the scientific study of the human brain, where dopamine is essential. This paper explores the potential of dopamine models to create a motivational decision-making system for robots operating in complex and dynamic environments. Dopamine, a chemical produced in the human brain, plays a crucial role in motivated behaviour by enhancing our desire to act, learning from past and new experiences, rewarding attention after observing the effects of our actions, and decision-making. Therefore, modelling the mechanisms behind dopamine secretion in robots enables the development of essential human traits that enhance their autonomy and performance [5].

The proposed method integrates principles from dopamine regulation [6,7,8] with reinforcement learning [9] methods to create a decision-making process that mirrors the adaptive and motivational characteristics observed in biological systems. By leveraging dopamine dynamics, this approach enables robots to make decisions based on immediate and delayed rewards, adapting their behaviour in response to changing circumstances and experiences. This biologically inspired model, therefore, offers a precise framework for generating autonomous behaviour in robots.

Our model is based on DopAct, which was proposed by Bogacz [6] in 2020. This model considers a brain-level simplification of dopamine production, dividing this process into three parts: dopamine secretion due to the execution of an action (eating if we are hungry or drinking if thirsty), due to habit formation (talking with a friend or eating our favourite food), and due to the difference between the expected value and the actual value based on learning by reinforcement (winning when gambling). The model shapes brain functions involved in decision-making, such as how the cerebral cortex is responsible for higher-level neural processes (such as perception of the environment) [10], the striatum role action selection [11], and the thalamus reward generation [12].

This research’s main contribution involves combining biologically inspired processes based on motivational theories with state-of-the-art dopamine models. We use the model to drive the Mini robot [13] to autonomously decide the best action selection strategy to maintain an optimal internal state. We define Mini’s internal state as a set of processes that emulate internal human functions: feeding, moisturising, sleep, and entertainment. These functions temporally evolve in the robot, creating internal deficits that change dopamine levels, urging behaviour execution to restore internal well-being. The agent’s goal is to autonomously reduce them to maintain its internal state in the best possible condition.

The results show how the model generates a dynamic internal state in the robot by learning to produce dopamine levels that predict and anticipate the rewards obtained after executing actions. We represent four simulated scenarios and a real case study with the Mini robot to show the adaptive and anticipatory mechanisms generated by the model. The dopamine levels drive decision-making in a social context by increasing dopamine levels when the robot presents internal deficits and when necessary stimuli are perceived. The dopamine levels related to each action are evaluated every time step by the Mini robot to select the most appropriate action to reduce its internal deficits.

In Section 2, this paper presents the background of this work. It describes the role of dopamine in the human brain and how it drives motivated behaviour. Moreover, the section presents dopamine models that emulate how this substance drives human behaviour. Section 3 describes the materials and methods used to generate autonomous behaviour in autonomous social robots. Section 4 shows the paper’s results, emphasising how dopamine levels adapt and anticipate different situations and how the Mini social robot employs this knowledge to behave autonomously. Section 5 and Section 6 enumerate the main limitations and potential future work. Finally, Section 7 concludes this work by highlighting its main findings.

## 2. Background

### 2.1. Dopamine Role in the Human Brain

Dopamine action as a neurotransmitter was discovered in 1958 when Carlsson and Hillarp [14] observed their role in cognitive brain functions like movement. Some years later, Crow et al. [15] discovered in 1971 that, by stimulating the ventral tegmental area in rats’ brains (similar to human brains), it was possible to motivate them to act. This finding demonstrated that some mammalian brain regions use dopamine to stimulate motivation and drive behaviour.

Later, in 1978, Wise et al. [16] related the ventral tegmental area and dopamine, perceiving that if dopamine levels decrease, the motivation to perform actions decreases too. This idea ends up giving a name to the pleasure hypothesis called hedonism [17], which defines dopamine as a mediator of pleasure-seeking.

The initial findings by Wise suggested that dopamine only played a crucial role in pleasure. However, later studies by Simansky et al. [18] in 1985 and Blackburn et al. [19] in 1989 proved that dopamine also influences the anticipation of obtaining rewards after task performance. For example, in the work by Blackburn et al., dopamine levels in rats are higher when the food stimulus is presented than when the food is ingested. Therefore, dopamine has an anticipatory character that boosts learning and motivation.

A recent study [7] showed that human dopamine levels change not only in response to stimuli before a reward but also after it. This discovery highlights the importance of dopamine in more than just anticipating rewards; it also plays a crucial role in processing the outcomes. As a result, researchers study dopamine to understand its role in driving motivated behaviour and guiding reward-based learning, being of great interest to neuroscientists of multiple disciplines, such as autonomous agents.

### 2.2. Biologically Inspired Dopamine Models for Autonomous Agents

This section examines works that use dopamine for decision-making processes in artificial agents. Some of them have been integrated within robotic systems, aligning with the objectives delineated in this project. We emphasise using reinforcement learning strategies, which are essential in most models to mirror the dopamine-driven mechanisms observed in the human brain. These mechanisms are fundamental in assimilating past experiences.

The work by Schultz et al. [20] from 1998 is recognised as a reference in the study of dopamine generation. Their model posits that dopamine secretion balances the moment we receive a reward after acting and the expectation we make of the same situation in the future. The authors define equations characterising dopamine level changes. However, this model is only a theoretical framework with complex but precise equations that have not tested a real application. Its application to real-time environments can be challenging due to its computational needs.

In 2009, Hayashi et al. [21] proposed an early investigation to simulate dopamine secretion within robotic systems. They use a predefined second-order equation to model the dopamine secretion response to various stimuli the robot perceives. This model modulates the Conbe I robot’s behaviour to simulate motivated behaviour and emotional states through dopamine secretion. This model contributes to the field of using dopamine to regulate motivated behaviour and decision-making. The model only uses a second-order equation to compute dopamine changes. However, it is limited by its static nature, lacking the capacity to evolve learning over time, thereby rendering dopamine levels strictly reactive.

In 2013, Baldassarre et al. [22] developed a neuronal model that derives from empirical activation functions of different brain regions to simulate dopamine secretion. This model was applied to a simulated humanoid robot iCub interacting with a mechatronic board [23]. The model showed accuracy in shaping dopamine influence on important brain functions such as motivated behaviour but lacked learning and anticipatory mechanisms.

Krichmar et al. [24] explored neural network-based dopamine and serotonin secretion models, implementing these in an iRobot to navigate. Their decision-making system dynamically selects the most appropriate response based on the levels of these neurotransmitters. However, the model only solves easy goal-directed tasks using a simple model that does not consider important dopamine dynamics such as pleasure or anticipation.

Some researchers have opted to model broader brain functions rather than focusing exclusively on neuronal specifics. For instance, in 2010, Friston et al. [25] introduced a behaviour generation model grounded in the free energy principle, encapsulating various brain function models in decision-making scenarios. This model evaluates the difference between perceived and anticipated stimuli, suggesting a system that aims at reducing disorder. Similarly, in 2019, Miller et al. [26] proposed a model that mimics brain learning processes, distinguishing between goal-oriented and habit-based action selection mechanisms. Although the model details dopamine generation very well, essential factors such as learning and anticipation are absent.

In 2020, Bogacz [6] proposed a novel dopamine model based on Bayesian analysis to elucidate the interaction between different actions and outcomes. The model emphasises the brain’s endeavour to minimise expected discrepancies between stimuli and outcomes and extends Schultz’s initial proposition by incorporating decision-making processes as a factor influencing dopamine secretion.

Drawing on the insights from Schultz et al. [20] and Friston et al. [25], particularly regarding the interplay between expected and actual rewards, we propose an updated version of Bogacz’s model [6] for dopamine generation in autonomous robots. Decision-making processes are managed by a high-level model, focusing primarily on the motivational aspects driven by dopamine. Similar to Krichmar et al.’s [24] approach with the iRobot, decision-making is orchestrated by a higher-level controller, which selects actions based on varying dopamine levels. The principles of these theoretical models have been combined and updated with other robotic models to incorporate adaptation, anticipation, and learning.

### 2.3. Autonomous and Adaptive Behaviour in Social Robotics

Emotional and motivational states have influenced biologically inspired autonomous systems using decision-making processes. Starting with Cañamero’s work [27], which introduced actions driven by needs and emotions in a simulated newborn, the field has advanced to incorporate complex human-like processes into robotic systems. Cañamero shaped motivation and emotion from a neuroendocrine perspective, adding specific hormones like dopamine and epinephrine to drive behaviour and regulate action exploration.This framework contributes to this work by defining the principles of biologically inspired behaviour with easy-to-implement equations based on simple dynamics. However, dopamine does not influence motivated behaviour, so the model does not consider learning and anticipation.

Reinforcement learning and cognitive functions, as seen in the works by Gadanho [28] and Malfaz et al. [29], marked early efforts to enable robots to adapt through interactions with their environments. They highlight the role of emotion in expressive behaviour but not the emotional role in decision-making. This idea was further developed by Lisetti et al. [30] and Ushida et al. [31] integrating emotional intelligence into social interactions. They emphasise the importance of emotional expressiveness in human–robot interactions. These authors address bio-inspired motivated behaviour by generating robot expressions and evaluating them with people. Action selection depends on predefined rules that link stimuli and actions, forgetting the dynamic and anticipatory mechanisms of human motivation.

Recent research explores the modelling of psychological states like stress and pleasure in decision-making. Cañamero et al. [8] and Lewis et al. [32] designed control systems that consider both internal states and external stimuli. Similarly, Hiolle et al. [33] designed an arousal-based controller for social robots based on pleasure and Lones et al. [34] a hormonal framework for autonomous agents in dynamic virtual worlds. These examples include hormones and neurotransmitters that generate internal biologically inspired processes in the robot. Although no names are provided, the effects of the substances suggest that dopamine is one of the most important.

Recent developments design robots that operate more independently, with circadian rhythms and internal deficits modelled for applications in caregiving and education. For instance, the results shown in the works of O’Brien and Arkin [35], and Egido-García et al. [36] provide new trends toward multimodal engagement and dynamic updating of biological factors for decision-making, highlighting the important role of long-term behaviour and learning from past experiences.

Multimodal systems like the one proposed by Hong et al. [37] and the hormonal decision-making system by Maroto-Gómez et al. [38] point to a future where robots can offer more personalised and effective support, emulating complex cognitive and physical functions. The transition from emotionally and motivationally driven models to robots capable of human-like interaction underscores the field’s aim to create systems that can navigate the complexity of human emotions, motivated behaviour, and social contexts.

Table 1 summarises the previous works, their challenges, and how the model presented in this work solves their most important limitations. This review highlights that most biologically inspired models for autonomous robots lack adaptive, anticipatory, and learning mechanisms to operate in complex environments, but some theoretical models provide a precise definition of dopamine to overcome these limitations.

This work is influenced by the models by Cañamero [27], Lewis et al. [32], and Hiolle et al. [33], which provide general simple equations to define motivated behaviours without focusing on the necessary role of dopamine regulation. Gadanho [28] combined complex computational learning and adaptive methods for autonomous robots but lacks anticipation. Finally, the work by Hong et al. [37] presents a precise definition of multimodal interaction by reacting to ambient stimuli without bio-inspiration. We combine these works with accurate and more complex dopamine models to solve challenges using Bogacz’s ideas [6] and reinforcement learning principles by Sutton and Barto [9].

## 3. Bio-Inspired Dopamine Model for Autonomous Robots

This section describes the materials and methods used to conduct the research presented in this paper. We first introduce Mini, the robot that integrates the dopamine model for decision-making. Then, we state the model features and how they generate dopamine based on the stimuli the robots perceive in their internal processes and the effects each action causes on the robot’s internal state.

### 3.1. Mini Social Robot

**Figure 1:**
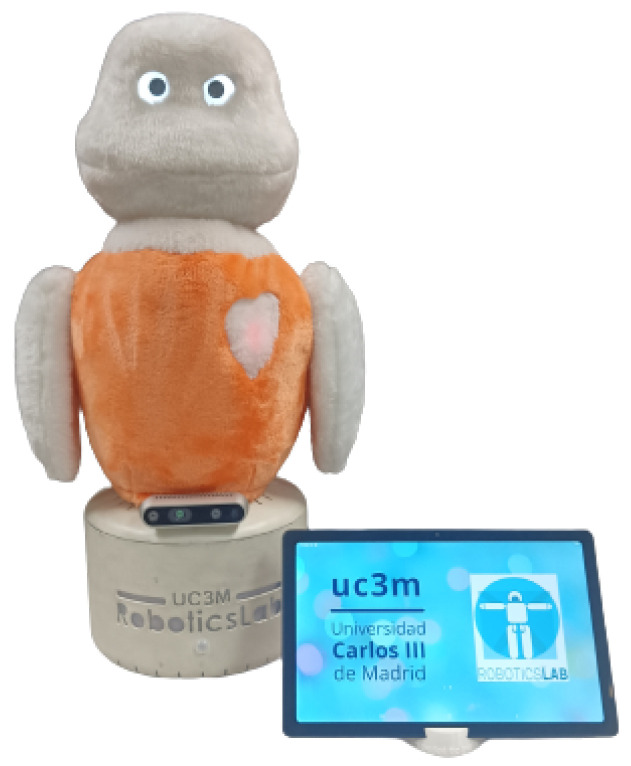
Mini, the social robot where the dopamine generation model has been integrated.

Mini [13] is a social robot designed by the Social Robotics Lab at the Universidad Carlos III de Madrid. It features a friendly appearance, as depicted in Figure 1 for human–robot interaction. It assists and accompanies older adults in private homes, day centres, and residences to improve their quality of life. The robot engages users through multiple actions, including entertainment, cognitive and physical stimulation, playing music, displaying photos, and monitoring the well-being of older adults.

Mini has touch sensors on its shoulders and belly to perceive when the user touches it and grabs its attention. A camera lets the robot perceive environmental stimuli, such as user presence or objects around. A microphone facilitates speech detection and communication with the user. Additionally, Mini incorporates a photosensor on its head to gauge ambient light intensity and regulate its activity and an RFID reader to perceive fictitious objects that simulate food (e.g., broccoli) or drink (e.g., water).

Regarding actuation capabilities, Mini features LEDs in its mouth, cheeks, ears, and heart, illuminating different expressions. The robot has five degrees of freedom in the hip, arms, neck, and head, enabling lively expressions. Two screens simulate eye movements, including changes in pupil size and blinking, reflecting the robot’s emotional states. Finally, Mini uses a speaker to play sounds and generate speech from text.

Mini integrates a tablet to display images, videos, and audio. It encourages interaction by facilitating various actions and collecting user responses to the robot’s inquiries. Mini exchanges information with its environment through this combination of sensors and actuators, allowing for adaptation to different situations.

Mini is a research social robot used to investigate how to generate biologically inspired behaviour in autonomous agents. One of the research lines we are working on is endowing the robot with animal-like processes so the user can take care of it by feeding it with food, moisturising it with drinks, and entertaining it together by playing different activities. We included sleep to avoid continuous interaction and regulate the robot’s activities. Therefore, we designed a software architecture integrating these biological processes to investigate how to generate autonomous behaviour with anticipation, learning, and adaptation.

### 3.2. Software Architecture

**Figure 2:**
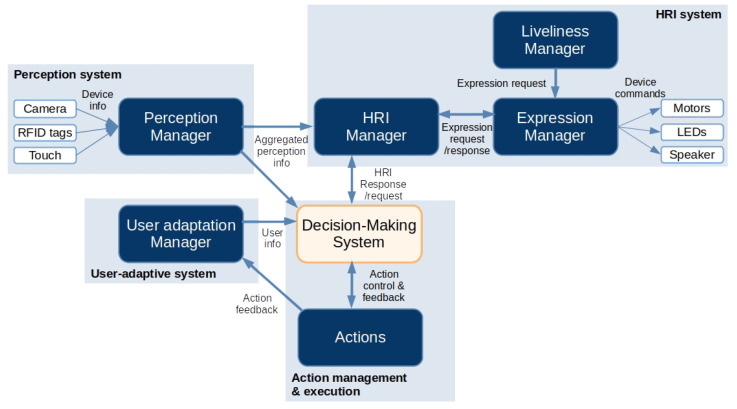
The software architecture of the Mini robot consists of several modules. The perception manager reads sensor information and generates homogeneous perception information for the other modules. The HRI system manages the interaction with the user and the environment, generating appropriate expressions and producing a coherent and adequate interaction. The decision-making system decides the best action in every moment based on the bio-inspired dopaminergic module and the information generated from the User-adaptive system and the perception manager.

The dopamine model to generate biologically inspired decision-making in autonomous robots belongs to the software architecture of the Mini social robot. Before describing the method proposed in this contribution, it is necessary to outline the main modules in the software architecture to understand how the model integrates it to yield autonomous behaviour. Figure 2 shows the software architecture highlighting the decision-making system, which incorporates the dopamine model. The architecture has been developed under the robotic middleware ROS to facilitate process communication. Reference [13] provided further details about the specific role of each module in the architecture. Next, we provide an overview of their principal functions and role in generating biologically inspired behaviour.

#### 3.2.1. Perception Manager

The perception manager is a module connected to the robot’s sensors (e.g., camera, RFID tags, or touch sensors) that receives raw data, processes it and generates a uniform message that the other modules can understand. This manager is responsible for informing the HRI manager of any environmental changes detected by the sensors that could impact the interaction process. Likewise, the perception manager communicates with other modules in the architecture, such as the decision-making system, indicating that the user will interact with the robot to drive actions that involve the robot and the user.

#### 3.2.2. HRI System

The HRI system manages the communication with the user, connecting the information provided by the robot’s sensors (including the interaction features) with the robot’s actuators to generate expressions and interactions. It consists of two main modules:

- Human–robot interaction (HRI) manager: Controls the communication between the robot and the user. This manager organises the interaction with the user, avoiding conflicts that may occur if several actions/modules want to use the same resources. This module mainly communicates with the actions and operates between the perception manager and the expression manager.
- Expression manager: Sequences the robot actuators depending on the expressions the HRI manager sends. It manages the commands sent to each actuator to ensure the robot executes the requested expressions. Moreover, it receives spontaneous expressions via the Liveliness manager to generate lively behaviour.

#### 3.2.3. User-Adaptive System

This module adapts the robot’s behaviour to the user features to improve and personalise the interaction. It comprises modules responsible for collecting user information to generate a dynamic profile for each user. It is divided into the user profiling manager and the user profiling action. The former receives information from the HRI manager about the actions performed by the user to generate interaction metrics that are lately used for the interaction. The latter requests information from the user to store it and update their profiles, which are saved and loaded in configuration files. The user profile is updated every time the perception manager detects that a new user is ready to interact with the robot using the camera with face recognition algorithms or RFID tags.

#### 3.2.4. Liveliness Manager

Social robots designed to assist people must incorporate lively behaviours that seem natural. This module generates commands depending on the robot’s state, such as blinking, pupil size, motor activation, breathing, and heart rate, to control the robot’s expressiveness. The expression manager receives these expressions and sends the commands to the actuators if there are no conflicts with previous expressions under execution.

#### 3.2.5. Actions

Actions are functionalities that the robot can execute, like games or cognitive stimulation activities. The decision-making System manages the activation of the actions depending on the robot’s purpose. They can be started, paused, resumed, or stopped at will to generate autonomous behaviour according to the robot’s situation. Some examples of the robot’s actions are multimedia playing or simulating biologically inspired actions like eating, drinking, sleeping, or playing to lead the user to use the robot frequently.

### 3.3. Decision-Making System with Dopamine Regulation

The decision-making system defines the motivational state of the robot imitating human biological processes. Depending on the robot’s internal and external situation, this module computes the intensity of the motivation to execute each action and selects the motivation with the highest intensity at every time step. Then, the decision-making system executes an action matched to the motivation with the highest intensity. Table 2 contains a glossary of technical terms to facilitate the understanding of the model dynamics.

**Figure 3:**
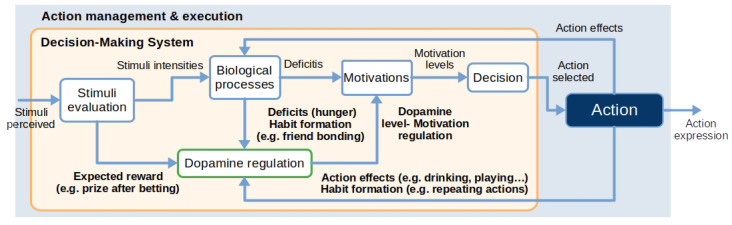
Dopamine biologically inspired model and its integration into the decision-making system. Dopamine levels depend on the reward we expect to obtain after perceiving stimuli like palatable food or executing an action that might produce a reward, like gambling. Internal deficits also raise our dopamine levels, leading us to reduce them with action execution. An example of this is drinking when we are thirsty. Finally, positive habit formation, like meeting friends, promotes dopamine generation and social actions. Dopamine levels regulate our motivation and affect our decision-making and behaviour.

The decision-making system mimics how human behaviour emerges by simulating how dopamine levels affect motivation in the human brain. Dopamine levels change depending on the stimuli we perceive, our actions, and how these actions affect the biological processes in our body (for instance, how eating reduces hunger). As Figure 3 shows, the decision-making system internally consists of a stimuli evaluation, the emulation of biologically inspired processes, a dopamine model to regulate motivation, and a decision stage to select the most appropriate action depending on the highest motivation.

The decision-making system emulates biological processes to drive action execution via motivation to maintain the robot’s biological processes in the best possible condition. Our Mini robot has four biologically inspired internal processes: sleep, feeding, moisturising, and entertainment. We use the homeostasis theory [39] to shape the evolution of internal processes. This theory has been previously used in related work [8,32,34,38], stating that living organisms maintain a stable internal environment despite changes in external conditions. This idea involves regulating various biological parameters, such as sleep or feeding. Homeostasis is achieved by detecting body imbalances from an ideal value and triggering actions to restore balance. For example, after a long time without drinking, our body detects water imbalance, motivating us to drink water and restore such imbalance. This idea is extended with four biological processes with the following function:

- Sleep: This process has been selected to regulate the robot’s activity/inactivity, avoiding fatiguing the user with continuous interaction.
- Feeding: This process regulates the hunger of the robot and has been selected to involve the user in the interaction taking care of the robot.
- Moisturising: Similar to feeding, this process involves the user in the interaction by fostering the user taking care of Mini.
- Entertainment: This process regulates user-robot interactions by playing different games.

The four biological processes autonomously promote and regulate human–robot interaction by requesting the user to take care of the robot and play with it, regulating the activity periods. These processes linearly evolve with time in the range from 0 to 100 following different variation rates ($vr_{i}$) depending on their importance in the robot and the nature of the process. Therefore, Equation (1) expresses each biological process’s value ($cv_{i}$) calculation in the time step *t*.

(1) $$cv_{i}\left(t\right)=cv_{i}\left(t-1\right)-vr_{i}$$

Biological processes have different ideal values ($iv_{i}$) at one range limit of 0 or 100. Sleep pressure accumulates throughout the day, with its optimal value being 0, as the body seeks to minimise this pressure by sleeping. Feeding levels start at 100 when we are satiated but decrease as hunger builds, prompting the need to eat and restore this value. Similarly, moisturising is ideal at 100 when fully moisturised but declines over time, leading to thirst and the drive to drink. Entertainment follows this pattern, where in the absence of stimulating activities, levels drop from the ideal value of 100, and boredom sets in, motivating us to seek entertainment to restore balance. We designed a system where feeding and moisturising evolve faster than sleep and entertainment since they are more important to the robot. Consequently, their variation rates are higher.

These processes evolve with time from their ideal value, provoking internal deficits in the robot that act as a detection mechanism of body imbalance. This means the robot must correct this internal imbalance by executing appropriate actions. Based on the robot’s biological processes, Mini can present four deficits: fatigue, hunger, thirst, and boredom. Deficits ($d_{i}$) are the difference between the current value of the process ($cv_{i}$) and its ideal value, as Equation (2) expresses. Table 3 shows the variation rates of each process and their associated deficit.

(2) $$d_{i}\left(t\right)=|iv_{i}-cv_{i}\left(t\right)|$$

The robot has to execute actions that restore its internal deficits, setting biological processes closer to their ideal value and maintaining the best possible internal condition. The biological model we propose considers four actions to fulfil this function: sleep, eat, drink, and play. The robot executes these actions in the following manner:

- Sleep: Mini closes its eyes, enters a relaxed mode without moving too much, and occasionally snores by performing different sounds. The action is executed if it perceives the light is off using a photosensor.
- Eat: Mini plays sounds simulating it is chewing food. The action is executed if it detects a broccoli object using an RFID reader.
- Drink: Mini plays sounds simulating it is drinking. The action can only be executed if the robot detects a water object using an RFID reader.
- Play: The robot performs different entertainment activities that are randomly chosen. Playing is only possible if the robot detects the user in front of it using a camera.

Table 3 shows that each action reduces the deficit of a related internal process. Sleeping reduces the fatigue deficit in 8 units, eating and drinking restore the hunger and thirst deficits in 10 units, and playing reduces the boredom deficit in 6 units. These effects have been empirically set to give more importance to feeding and moisturising than sleeping and entertainment, considering that eating and drinking have a punctual impact while sleeping and playing are longer lasting.

Most of the time, the possibility of executing an action depends on the available specific resources. The robot can only act if a related stimulus is perceived and a moderate deficit exists. In this scenario, the robot can perceive fictitious broccoli and water objects designed as RFID tags using an RFID reader, the light intensity using a photosensor, and the user presence using the camera. Therefore, eating occurs if the food broccoli is available and the robot is hungry. Similarly, Mini can only drink if water is perceived and it is thirsty, sleep if the lights are off and fatigued, and play if the user is present and is bored. We have defined that a deficit is active only with a value above 20 units to avoid small deficits leading the robot to execute an action that is not necessary at all.

When a deficit is high enough, and the stimulus to reduce it is available, a motivation to drive the associated behaviour emerges. Since the robot can have multiple active deficits and simultaneously perceive different stimuli, more than one motivation can be active (e.g., motivation to play and sleep). However, the robot can only execute one action simultaneously, so the motivation with the highest intensity will be dominant and drive its associated behaviour. Dopamine levels affect each motivation depending on the robot’s situation. Therefore, each motivation has an associated dynamic dopamine level that informs the robot about its decision and action.

The relationship stimulus–deficit–motivation–action is unknown by the robot from scratch. The robot only knows each action’s effect and the process variation rates. Therefore, the proposed system learns and anticipates the dynamic conditions of the ambient environment to reduce its internal deficits and maintain the best possible internal state by executing appropriate actions. The system measures this situation with the well-being level (*Wb*), a metric that represents how good the robot’s internal state is by measuring the average value of the internal deficits ($\bar{d_{i}}$) from 0 (lousy well-being) to 100 units (ideal well-being) (see Equation (3)).

(3) $$Wb=100-\bar{d_{i}}$$

The dopamine regulatory mechanism used in this contribution drives the robot to select the best action in each situation, simulating a motivationally driven system. The dopamine model that we integrated into our decision-making system is a variation of Bogacz’s model, called DopAct [6]. DopAct considers a brain-level simplification of dopamine secretion. We selected it due to its low computational cost, good performance with random reward distributions (like the different rewards each action generates in our model), and bio-inspired modelling based on real human studies.

Our model generates and learns the dynamics of dopamine levels in response to rewards and predictions of rewards over time. The model relates actions with the different motivations the robot experiences to maintain an optimal state by simulating dopamine secretion in the human brain. The model considers three key ideas: dopamine secretion due to the execution of an action (eating if we are hungry or playing if we are bored), due to habit formation (talking with a friend or learning for promotion), and due to the difference between the expected value and the actual value based on learning by reinforcement (expecting a prize if we bet in the lottery). Due to the combination proposed in this paper of the dopamine model with the regulation of internal processes, we emulate the functions of three brain regions: the *cerebral cortex*, responsible for higher-level neural processes (such as the perception of the environment) [10], dopamine generation in the *ventral tegmental area* [40], and the *striatum* role in action selection [11].

The model uses different equations to calculate the reward the agent expects to obtain the reward (expected reward) after executing an action (action effect). The expected reward depends on the dopamine level generated for each motivation at each time step. The equations draw on temporal difference, a reinforcement learning method [9] that learns how an agent improves its predictions by comparing expected rewards over time, helping it make better decisions based on past experiences.

Equation (4) computes the dynamics of the expected reward ($\dot{v}$). This value depends on the values of a gain matrix ($\bar{w}$) that represents the temporal evolution of dopamine levels based on the past events experienced by the agent. The expected reward value in time step *t* is subtracted from the multiplication of the gain matrix by the stimulus matrix ($\bar{s_{v}}$), which indicates the time lapse when a stimulus is available. The derivative of the expected reward is scaled by a time constant (τ) that naturally regulates dopamine secretion, showing a subtle decay between dopamine peaks like in human studies. This equation exemplifies how we look for food in places where we already found it using the gain matrix ($\bar{w}$) to store information about past experiences and the stimulus matrix ($\bar{s_{v}}$) to know about the stimulus availability.

(4) $$\dot{v}=\frac{1}{\tau }\cdot \left(\bar{w}\cdot {\bar{s}}_{v}-v\left(t\right)\right)$$

Equation (5) computes the dynamics of the dopamine variation (${\dot{\delta }}_{vr}$) using the value of the total reward. The total reward considers the reward (*r*) obtained after executing an action plus the expected reward (*v*) obtained from Equation (4) minus the expected reward value at the previous time step (v(t−1)) minus the internally generated dopamine due to reward ($\delta_{vr}$) in time step *t*. This result gives the derivative of dopamine secretion scaled by a time constant ($\tau_{\delta }$) to decrease dopamine levels when the reward disappears. This equation represents how we learn to play video games. If we cannot move to the next level after repeating an action, we change our strategy to reduce the error we have committed by only repeating the actions that led us to success.

(5) $${\dot{\delta }}_{vr}=\frac{1}{\tau_{\delta }}\cdot \left(r\left(t\right)+v\left(t\right)-v\left(t-1\right)-\delta_{v}\left(t\right)\right)$$

Equation (6) updates the gain matrix ($\bar{w}$) that represents the temporal evolution of dopamine levels based on the past events experienced by the agent. The update considers a learning factor ($\alpha_{v}$) that balances the reward importance of new and past experiences, the prediction error ($\delta_{vr}$), and eligibility traces ($\bar{e}$) to reduce the potential impact of past experiences lived by the robot while increasing learning speed by lowering the learning factor ($\alpha_{v}$) as the robot gains new experience. In this equation, the eligibility traces make the robot forget experiences that occurred long ago, giving more importance to recent ones. For example, once we have learned how to ride a bike, we repeat actions that enable us to ride well, forgetting that we occasionally fall during the learning process.

(6) $$\dot{\bar{w}}=\alpha_{v}\cdot \delta_{vr}\cdot \bar{e}$$

Equation (7) updates the eligibility traces following Sutton and Barto’s [9] proposal. This update balances past experiences and combines them with stimulus availability by considering the sum of the retention factor of the eligibility traces (λ), a factor that reduces the impact of past experiences in dopamine secretion, multiplied by the past eligibility traces plus the stimulus matrix (${\bar{s}}_{v}$) minus the average value of the previous traces ($\bar{e}$). As occurs with the reward and dopamine values, the eligibility traces are weighted by the time constant ($\tau_{\delta }$).

(7) $$\dot{\bar{e}}=\frac{1}{\tau_{\delta }}\cdot \left(\lambda \cdot \bar{e}\left(t-1\right)+{\bar{s}}_{v}-\bar{e}\right)$$

The dopamine model integrates two novelties from psychological studies that Bogacz did not consider. First, it incorporates the reward update proposed by Cañamero and Lewis [8] using Equation (8). Like Cañamero and Lewis, dopamine levels increase with a pleasure value ($\delta_{vp}$) obtained after executing an action that reduces the robot’s deficits. In Equation (8), the pleasure obtained directly depends on how strong the deficit reduction ($\Delta d_{i}$) is, a scaling factor (*s*) that regulates dopamine secretion, a γ factor that considers the deficit value compared to its maximum possible reduction (Equation (9)), and a β weight that indicates the preferences of each user toward a specific stimulus. These preferences can be biological (e.g., a higher concentration of a particular type of taste buds) or sociocultural (e.g., eating food typical of a place). Finally, a constant value (*k*) is included to reduce dopamine levels within a specific time frame after obtaining a reward.

(8) $$\delta_{vp}=\Delta d_{i}\cdot s\cdot \gamma \cdot \beta -k,$$

where

(9) $$\gamma =1+\frac{d}{V_{max}}$$

From the dopamine reward Equation (5) and the pleasure Equation (8), we define the dopamine level ($\delta_{vl}$) in Equation (10) as the sum of both values.

(10) $$\delta_{vl}=\delta_{vr}+\delta_{vp}$$

Based on findings from Seitz et al. [7], the second innovation regulates dopamine levels to prevent an increase upon repeated exposure to the same stimulus. The robot remembers stimuli variations after a reward of 30 s to avoid computing rewards experienced in the same situation. For example, if food is perceived as the stimulus and the sequence after drinking water is recalled during the food search, it forms an inconsistent combination. Conversely, if the sequence after the stimulus is related to a meal, it is considered congruent. Both congruent and inconsistent combinations decrease motivation, with congruent ones having a more substantial effect. In the model, dopamine secretion decreases by a quarter for congruent combinations and half for inconsistent ones using the (ϕ) value.

Dopamine levels are also affected by the time difference between perceiving the stimulus and obtaining the reward. Thus, motivations targeting goals closer in time yield higher rewards due to quicker positive effects. This idea is applied by introducing a discount factor, η, proposed by Stelly et al. [41]. This factor ensures that the motivation to obtain the reward decreases to around 5% of its initial value after 10 min. Combining these concepts, we propose Equation (11) to incorporate the time difference and the discount factor typically used in reinforcement learning to calculate a regulated dopamine level $\delta_{vreg}$ from the reward and pleasure values. In this proposal, *RT* is when the agent obtains the reward, and *ST* is the time of stimulus perception. The regulated $\delta_{vreg}$ is the final value used in the following sections to represent dopamine levels in the robot.

(11) $$\delta_{vreg}=\delta_{vl}\cdot \eta^{\left(RT-ST\right)}\cdot \phi$$

Table 4 defines the model parameters and their values. They were selected based on the robot limitations, biological factors observed in studies described in Section 2, and Bogacz [6], Stelly et al. [41], Cañamero and Lewis [8], and Seitz [7] models.

We use a sampling time (dt) of 1 s, aligning with the update of the biological processes in the robot. Micro-state duration (Microt) defines time discretisation to compute gain matrices. It is established at 20 s (using Bogacz’s suggestion) to minimise matrix size in learning computation. This duration is chosen based on the reward time constant, set at 20 s, to ensure correct learning system dynamics. For learning variables, we aim for realistic and accurate performance. The learning factor ($\alpha_{v}$) and eligibility trace retention (λ) are 0.03 and 0.75, based on the original model. The reward time constant (τ) is 20 s, a value consistent with experiments, varying across individuals based on actions and arousal levels. These values have been empirically determined in the preliminary test.

Given the robot’s relaxed interaction with users and actions lasting a few minutes, we use a reward time constant (τ) of 20 s. The dopamine generation time constant ($\tau_{\delta }$) is selected 10 times lower, at 2 s, to avoid affecting much slower reward dynamics. Pleasure-related model parameters ensure a reasonable decay time of pleasure: 0.05 for the decay factor (*k*) and 0.1 for the scale factor (*s*).

## 4. Results

The results presented in this section aim to provide insights into how the dopamine model proposed in this paper learns to generate autonomous behaviour in biologically inspired robots with five scenarios. Next, we will review each scenario’s experimental setup and the results obtained for each case.

### 4.1. Experiments Setup and Conditions

We designed five scenarios to show the model performance, four in simulation and one using the Mini social robot. We trained the model using an Intel NUC computer with an Intel Core i7-1165G7 processor, featuring 4 cores and a clock speed of 2.8 GHz. The system includes a 16 GB of DDR4 RAM. It uses a 512 GB NVMe SSD for storage. The operating system is Ubuntu 20.04 LTS.

The software architecture and the dopamine model were developed using the robot middleware ROS 1 [42] to facilitate the communication of the different modules and the programming language Python 3 to implement the algorithms described above. We use the NumPy Python library for data management and Matplotlib to generate the results. The model uses the parameters defined in the previous sections summarised in Table 4. The model does not employ any dataset or pre-processing since reinforcement learning generates training data from scratch, obtaining experiences from interacting with the environment. The new knowledge is saved as NumPy (Python library) files with the extension .npz that can be loaded and transferred to the Mini robot.

Section 4.2 shows how dopamine programming anticipates future rewards. For this scenario, we ran the model in simulation for 150 rounds. We fixed the times when the robot perceived the stimulus at the 70-s mark and when the agent obtained the reward at 290 s to analyse how dopamine levels rise with the stimulus perception, anticipating that a future reward will be received. The results only show the first 100 rounds since learning converges at this point.

Section 4.3 shows the model’s performance when the stimuli and rewards are not perceived at the expected moment but randomly. In this scenario, the stimulus randomly appears between the 50 and 150-s marks, and the agent randomly obtains a reward between the 250 and 350-s marks. We used a normal distribution generator to generate the random stimulus and rewards. We trained the model in simulation for 100 rounds. Then, we ran two additional simulations with a random stimulus perception forcing the reward to be obtained before and after the 300-s mark to analyse how dopamine levels change depending on when the agent receives the reward.

Section 4.4 follows the same dynamics as the first scenario but compares how the system reacts when the robot interacts with a friendly and unfriendly user. The first case shows dopamine levels when the reward the user provides is positive (caress), and the second case shows dopamine levels when the user provides continuous negative feedback by hitting the robot. Both scenarios consider variable times to perceive the stimulus (between seconds 50 and 150), variable times to obtain the reward (between seconds 250 and 350), and introduce the novelty of considering variable amounts of rewards with caresses and hits having different intensities. We trained both cases for 100 rounds to compare the outcomes in the final round.

Section 4.5 shows the autonomous selection of different actions to maintain an optimal internal state using dopamine secretion as the precursor of motivated behaviour. We obtained the results by training the model for 1000 rounds. In this scenario, the four biological processes (feeding, moisturising, sleep, and entertainment) in the robot evolve with time, causing deficits (hunger, thirst, sleep, and boredom) following the dynamics in Section 2.3. The robot randomly perceives a stimulus (broccoli RFID, water RFID, user presence, or lights off) with a simulated 10% chance and learns to select the most appropriate action (eat, drink, play, or sleep) depending on the situation. We show how dopamine levels change depending on the situation and the moment the agent obtains the reward by adapting to the different conditions.

Finally, Section 4 shows how the Mini robot optimises the well-being metric using the trained model presented in Section 4.5. We ran the model for 300 steps, each 1 s, for 5 min, facing a user with the Mini robot. The user sat in front of Mini to test the model’s performance in a laboratory setting. During this time, the biological processes in the Mini robot evolved, causing deficits. These deficits required by the experimenter help to reduce them. Mini verbally informed the user about its internal deficits. The user could show broccoli and water RFID cards and give them to Mini to reduce hunger and thirst. An RFID reader in Mini’s belly detects these objects. When bored, the user’s presence detected by a camera motivates Mini to play with the user. Finally, if Mini was fatigued and lights were off (detected using the photosensor), it could sleep to reduce this deficit. Mini only requests reducing one deficit at a time based on its motivational state. Combining these autonomous behaviours, Mini regulates its well-being.

### 4.2. Anticipation

**Figure 4:**
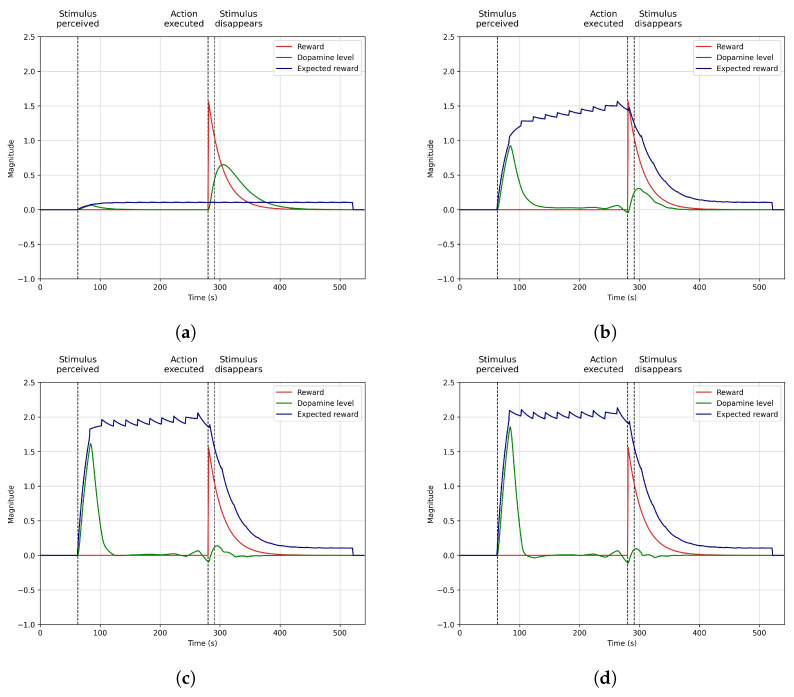
Dopamine regulation anticipate action selection when a stimulus driving a future reward is perceived. (**a**) Round 1. (**b**) Round 30. (**c**) Round 70. (**d**) Round 100.

Figure 4 the model’s performance in learning to anticipate the benefits of eating when the robot is hungry and perceives food (broccoli). It generates high dopamine levels upon perceiving the stimulus, which motivates the robot to perform the action of eating and subsequently obtain a positive reward. In this scenario, the robot always perceives the stimulus (broccoli) at the 70-s mark and the action of eating with the corresponding reward at the 290-s mark.

At the beginning of the learning process (Figure 4a), the robot does not know that eating broccoli reduces hunger, producing internal benefits since the robot has not experienced this event before. For this reason, when the stimulus is perceived at the 70-s mark for the first time, dopamine levels (in green) are shallow and do not motivate the robot to eat. However, if Mini consumes broccoli around the 290th s, it receives a high reward produced by the reduction of the hunger deficit, indicated by a surge in dopamine release immediately following the eating action. After consuming broccoli, dopamine levels exponentially decrease (by the action of the τ time constant), meaning broccoli is no longer available, and hunger has been reduced, so there is no need to continue eating.

As learning advances and the robot experiences the same situation many times (Figure 4b,c show rounds 30 and 70 of the robot experiencing this situation), the robot creates a habit and learns that broccoli restores hunger. Therefore, dopamine levels do not increase when eating but start significantly increasing right after perceiving broccoli. This means the system learns to anticipate the reward obtained when eating due to having a high internal deficit (hunger) and perceiving broccoli.

This effect motivates the robot to eat when broccoli is perceived, establishing a relationship between broccoli, hunger, the motivation to eat, and the action of eating. As Figure 4 highlights in blue with the expected reward plot, there is a delay between perceiving broccoli, eating, and obtaining the reward. This delay represents that sometimes the robot does not instantly receive the reward and is delayed in time because the stimulus is unavailable or the action cannot be executed at that moment. The expected reward increases during this time until it converges in round 100 (Figure 4d), indicating that the reward will usually be obtained around the 290-s mark. After this moment, the expected reward value starts dropping with a slope that depends on the τ time constant and the previous experiences lived by the agent (as Equation (4) represents).

The process depicted in this scenario shows how the model anticipates the reward obtention by learning that the habit of perceiving broccoli when hungry and eating produces positive internal deficits. This situation is possible thanks to the motivational system based on dopamine secretion that acknowledges this situation, producing high dopamine levels when it expects that a positive reward will be obtained. This scenario can be replicated in other robotics areas, like mobile robots, where anticipating specific actions while navigating to a goal could significantly reduce the time required to complete the task.

### 4.3. Dynamic Stimuli and Rewards

**Figure 5:**
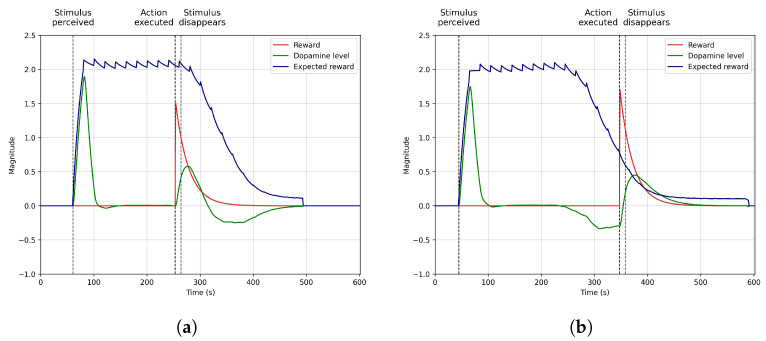
Obtaining rewards before or later than expected causes dopamine secretion to drastically vary its levels, affecting the learning dynamics of future anticipation. (**a**) Reward obtained before than expected. (**b**) Reward obtained later than expected.

Figure 5 illustrates the dopamine evolution when the reward is obtained before (Figure 5a) and after (Figure 5b) the robot expects it. After completing 100 rounds of learning, we generate these two situations using the same situation as explained in the previous section but changing the moment the robot obtains reward, and the stimulus is perceived. We trained the system with variable stimulus perceptions between seconds 50 and 150 and random rewards between seconds 250 and 350. This scenario shows how the system anticipates dopamine secretion when a stimulus produces a delayed reward at random moments, correctly learning to map rewards to stimuli perception.

Figure 5a shows an example of dopamine evolution when the reward is obtained before expected. During training, the model typically generates rewards of around the 300-s mark, so the robot expects it at that moment. Dopamine significantly increases around the 75-s mark due to perceiving the stimulus, anticipating that a reward will be later obtained. This peak motivates the agent to execute an action and receive a reward before it is expected. This reward increases dopamine levels again around the 280-s mark. However, since the reward was typically expected around the 300-s mark but occurred before, dopamine levels decreased, indicating the agent did not obtain the expected reward on time. It is important to highlight that dopamine levels are not absolute but relative, so negative values are possible in the model as the dopamine evolution shows right after obtaining the reward.

Figure 5b shows the opposite case. The reward is obtained later than expected. In this figure, the agent perceives the stimulus around the 60-s mark. Since this stimulus typically produces a reward around the 300-s mark during the learning process, the system anticipates this event by causing a dopamine peak when perceiving the stimulus. This peak motivates the agent to behave and take the stimulus. Figure 5b shows that if the robot obtains the reward later than expected, dopamine levels decrease when the reward is expected due to the lack of reward. Later, around the 350-s mark, the reward is finally obtained, producing a dopamine peak that indicates the positive effects of the reward on the agent. Then, dopamine levels decrease to the baseline levels, reducing the agent’s motivation to behave.

This scenario shows the typical scenario social robots experience during human–robot interaction. The robot usually does not obtain the reward when expected since it depends on the user’s actions. Consequently, effectively learning to deal with these situations yields an important improvement in autonomous robots acting in dynamic environments.

### 4.4. Learning to Avoid Negative Situations

**Figure 6:**
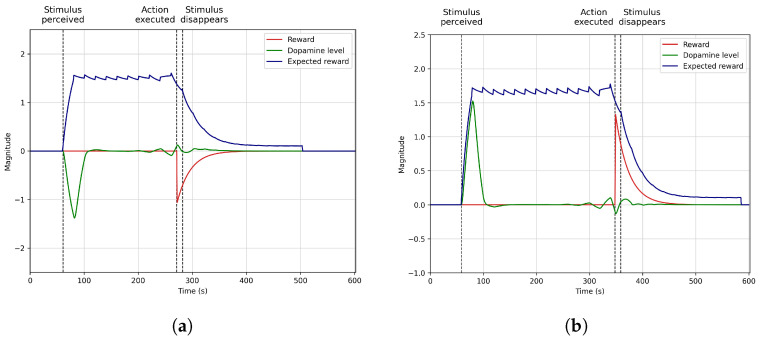
Interacting with different kinds of users drives dopamine levels to vary, leading the agent to avoid negative situations like being hit and promote positive ones like receiving a caress. (**a**) Learning to receive a caress when interacting with a friendly user. (**b**) Learning to avoid being hit when interacting with an unfriendly user.

Figure 6 compares the dopamine model performance when the agent faces two users with different social behaviours (friendly and unfriendly). This scenario varies when the agent perceives the stimulus, obtains the reward, and determines the intensity of the reward by considering caresses and hits with different intensities. On the one hand, Figure 6a shows how the agent learns to anticipate that a friendly user will provide positive affective support by caressing the robot. When Mini perceives the user, dopamine levels increase, motivating the robot to start a social interaction since, in previous encounters, this user repeatedly caresses Mini.

On the other hand, Figure 6b shows how the model learns to avoid interacting with an unfriendly user who continuously hits the robot. This time, dopamine levels substantially decrease when perceiving the user since the robot receives a hit every time they meet. This situation reflects how the model deals with continuous negative rewards driving the robot to avoid certain situations.

The expected reward line (blue) is similar for both cases since the robot expects to receive a reward independently of the kind of user the robot faces. This fact indicates that the system correctly anticipates the behaviour of both types of users but produces different dopamine levels depending on the behaviour the user performs.

### 4.5. Learning Autonomous Action Selection

**Figure 7:**
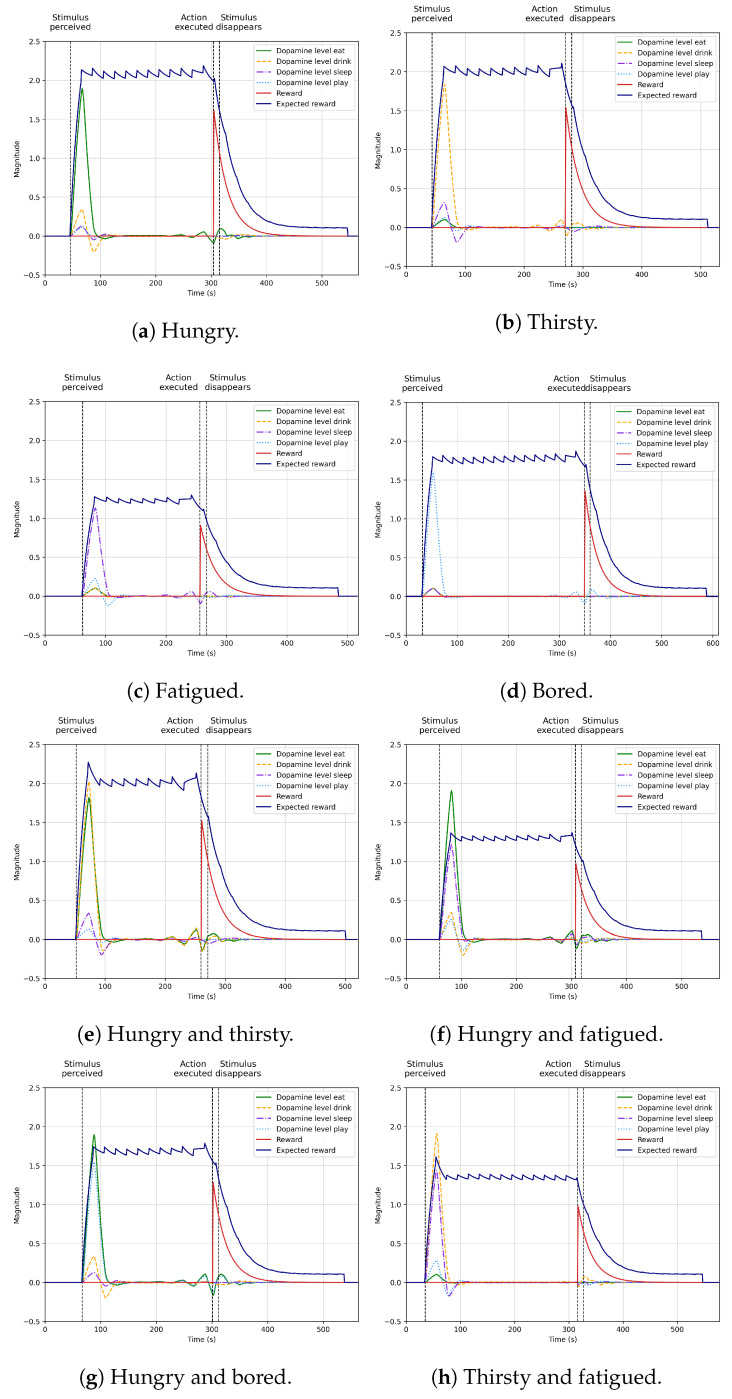

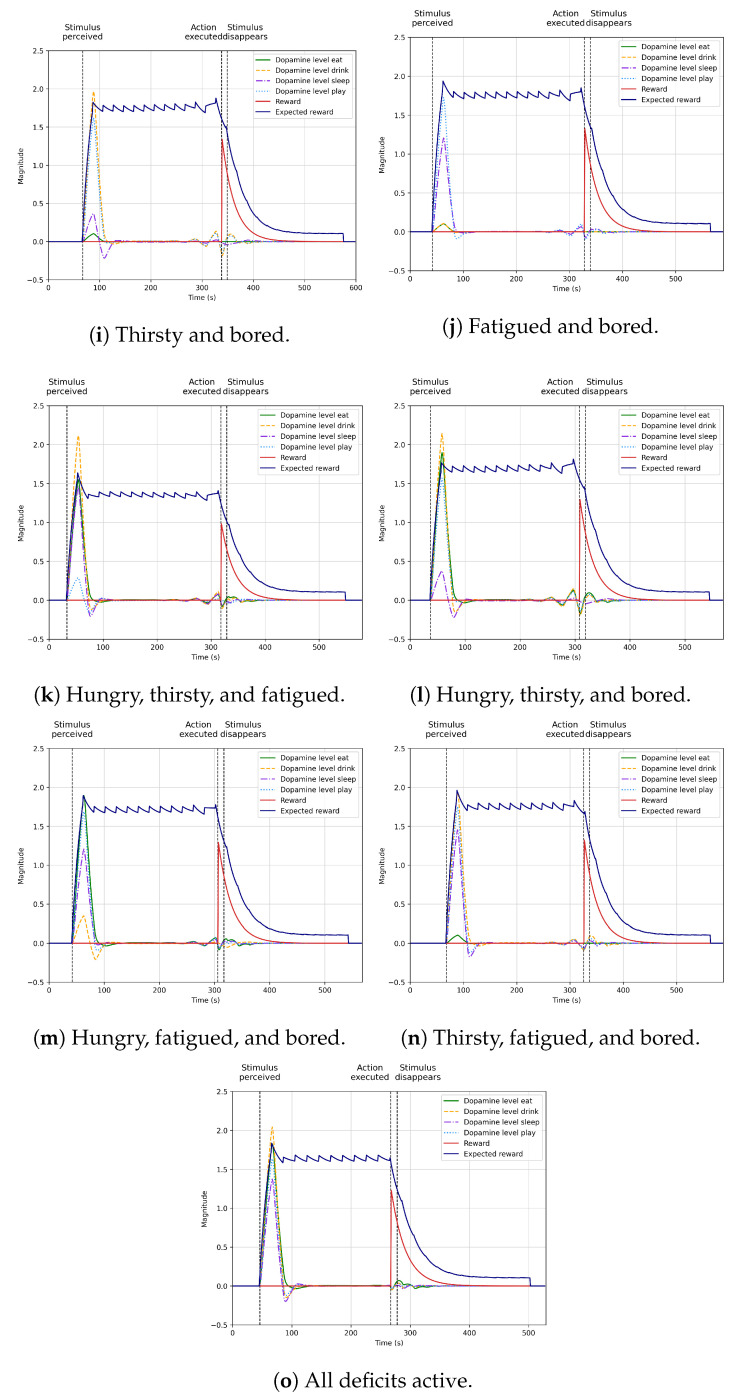
Learning results for each situation (hunger, thirst, sleep, boredom, and their combinations) and actions (eat, drink, sleep, and play) the robot experiences.

Figure 7 illustrates the learning results for the scenario presented in Section 2.3. The scenario features Mini with four internal processes (sleep, feeding, thirst, and entertainment) whose temporal evolution leads to four internal deficits (fatigue, hunger, thirst, and boredom). The robot has four actions (sleep, eat, drink, and play) to reduce internal deficits. Mini can only execute actions if there is a considerable internal deficit and perceives necessary stimuli (lights off, broccoli, water, and the user) to complete the action. In this scenario, the stimuli and the rewards appear randomly, ensuring that the reward will always come at least 180 s after perceiving the stimuli.

The agent’s goal is to learn the relationship among stimuli–deficit–motivation–actions using the dopamine system to anticipate the effects of stimuli and action execution on the internal processes. This goal considers the possibility that more than one deficit and stimuli are active simultaneously, leading the robot to learn which action is the most important at each moment, depending on the situation. The subplots in Figure 7 show the dopamine levels attached to each motivation (motivation to sleep, to eat, to drink, and to sleep) if the necessary stimulus is perceived, combining the different deficits the agent can have.

Figure 7a–d show the learning results when the robot presents only one deficit alone (hunger, thirst, fatigue, and boredom). The robot correctly learns which action to execute in these figures when there is only one active deficit. Figure 7a shows that the best action is eating when hungry since the dopamine levels for eating significantly provide the highest secretion rate. Figure 7b shows that when thirsty, the best alternative is drinking, Figure 7c that the best alternative is sleeping when fatigued, and Figure 7d that the best option is playing when bored. The learning system positively anticipates the reward obtained, segregating dopamine after perceiving the stimuli.

Figure 7e–j show cases where the robot simultaneously presents two deficits. For example, the robot is hungry and bored or thirsty and fatigued. In these situations, the robot correctly learns to anticipate the reward obtained when it perceives the associated stimuli. The difference in these situations is that if, for example, the robot is hungry and thirsty simultaneously and broccoli and water are perceived, the learning system has to learn how to deal with this situation. As displayed in Figure 7e, in this situation, the robot learns that drinking and eating are the best actions, with the dopamine secretion related to drinking slightly above dopamine levels for eating. The subtle difference in this case is due to the random component of the model since the feeding and drinking internal processes have the same importance for the robot.

If we analyse another case, for example, when the robot is hungry and bored (Figure 7g), we can see notable differences between the dopamine secretion for eating and playing. Dopamine levels for eating are significantly higher than for playing since reducing the hunger deficit is more important for the robot than reducing boredom. This result is due to the model definition but demonstrates how the system correctly learns to reduce internal deficits, prioritising those more critical for the robot.

Figure 7k–o show the results when the robot has three or four deficits simultaneously. In all these cases, the robot correctly learns to reduce internal deficits, giving more importance to reducing hunger and thirst than fatigue and boredom. The robot can exhibit autonomously biologically inspired behaviour to correctly maintain an optimal internal state, anticipating future expected rewards. Based on the results obtained in these situations, we show how the dopamine model correctly learns which action is the best option in each situation, learning to reduce the agent’s internal deficits correctly.

### 4.6. Integration into the Robot

**Figure 8:**
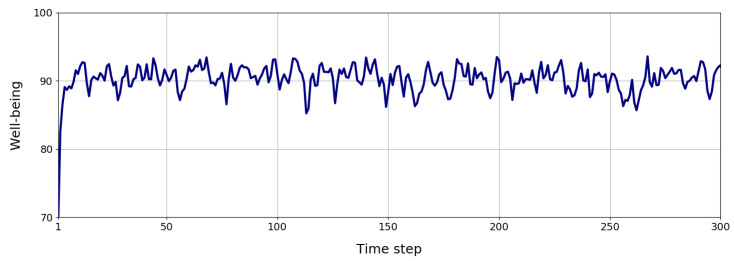
Well-being regulation during 300 time steps using the dopamine mechanism to select the best action in each situation autonomously.

Figure 8 shows the well-being regulation of the biologically inspired internal processes integrated into the Mini social robot to exhibit autonomous behaviour during 300 time steps. The action selection method based on dopamine secretion allows Mini to maintain a well-being value of around 90 units of 100 during its lifespan. Although the deficit of all processes had an initial value of 50 units, which means a well-being value of 50 units, the robot can improve it with time by restoring its internal milieu. The graph evolution shows that occasionally, the well-being value drops, meaning that the robot’s internal deficits are increasing, and the robot is hungry, thirsty, bored, or fatigued. This situation motivates the robot to take action and correct the active deficits. Once the agent corrects the deficits, the well-being value increases again, improving the agent’s condition.

This result demonstrates how the robot can maintain an optimal internal state, reducing its internal processes properly. However, since it can only reduce one process simultaneously, its well-being value cannot attain the perfect state (100 units) since while one process improves, the others worsen. This scenario shows that the model training in simulation can be integrated into a real robot and tested in a natural environment. Moreover, the processes involved in managing the robot’s internal state can be extended to generate more diverse behaviours, allowing the robot to complete other tasks such as social interaction or navigating the environment to look for resources. These results highlight that dopamine models enable autonomous robot behaviour by including features essential to performing well in complex environments: adaptation, learning, and anticipation.

## 5. Limitations

The work presented in this paper has some limitations that we should address in future updates. These limitations are principally related to the dopamine model parameters and features.

- Time discretisation: The model by Bogacz [6] faces challenges in learning due to the discretisation of time into states. Variations in dopamine generation occur when rewards appear between microstates, stemming from the model’s reliance on a single value per microstate in the primary gain matrix.
- Computational resources: Dopamine models presented in Section 2 with an accurate representation of dopamine secretion in the human brain require complex equations that need moderate computational resources. This limitation can be stressed if more stimuli and biological processes act in the model since the number of combinations the robot has to learn significantly increases. Consequently, computational resources can be problematic if the model increases in size.
- Empirical parameters: The model proposed in this paper depends on many parameters that affect its performance. We have selected their values based on the original models and an empirical evaluation conducted to obtain a specific robot behaviour that prioritises some processes (e.g., feeding) above others (e.g., sleep). However, factors such as the time constant, agent reaction speed influence, and reward-related parameters strongly impact the resulting robot behaviour, and more tests comparing different configurations might contribute to a better understanding of the model dynamics.
- User evaluation: Robots work to assist people in many different applications. Consequently, robot users must test these systems before we deploy them in real scenarios. Mini is a robot dedicated to older adults’ healthcare, acting as a system for conducting cognitive stimulation activities while entertaining the user. The biologically inspired model shapes four biological processes intended for the user to take care of the robot, so evaluating how people perceive Mini’s behaviour in terms of diversity or enjoyment would be of interest to continue exploring this research line.
- Ethical considerations: The development of bio-inspired decision-making systems in robots designed for human–robot interaction yields several ethical considerations that must be addressed. These systems, such as those modelled after dopamine-driven behaviours, may unintentionally replicate human biases, leading to unfair or discriminatory decisions. Additionally, collecting personal data to enhance robot behaviour raises significant privacy concerns, necessitating robust data protection and transparency. Moreover, as robots become more human-like in their decision-making, the nature of human–robot interactions could change, potentially blurring the distinction between machines and living beings. This could lead to ethical challenges related to autonomy, consent, and the treatment of robots. Therefore, it is crucial to carefully consider these factors to ensure that bio-inspired robots enhance human–robot interactions fairly, transparently, and ethically soundly.

## 6. Future Work

Our future work related to this research line is mainly associated with its evaluation of people, testing the system in more complex environments, and different applications. Next, we will describe the future work of this study.

- Model dynamics: Further exploration of dynamic parameter determination based on real-time factors could refine the model’s responsiveness, especially concerning the simplification of dopamine secretion dynamics. The scenario we propose consists of configuring different parameters and testing how they influence the robot’s behaviour. For example, we want to test if changing the variation rates of the biological processes affects user engagement with the robot since a psychological theory states that a high frequency of actions might increase user engagement in social scenarios. Similarly, by changing the importance given to new experiences over past experiences, we can evaluate the robot in personalising the selection of entertainment activities based on user performance and preferences.
- Role of pleasure: Pleasure plays a very important role in dopamine secretion. This study considers that dopamine increases when the robot consumes broccoli or drinks water. However, other types of food, drinks, or entertainment activities can produce different dopamine pleasure levels. Therefore, defining a robot with different preferences (for instance, preferring chocolate over broccoli) can be of interest to motivate users to discover these patterns and engage them with the robot.
- Testing in more complex scenarios: The scenarios we propose aim to evaluate the model’s performance and analyse its possibilities. However, further tests involving more biological processes such as social needs (talking, affect, or physical interaction) with more stimuli (caresses or different user responses) might produce a more comprehensive and meaningful robot behaviour that can operate in more challenging environments.
- Different robotic applications: This paper presents the application of the dopamine model to social robots interacting with people in an entertainment scenario. However, we want to explore the model’s performance in other scenarios like mobile robotics, where a motivational system based on dopamine, as proposed in this contribution, can promote environmental exploration, yielding interesting situations. For example, the robot’s exploration can be regulated by curiosity, a psychological factor highly affected by dopamine [7].
- Exploring other chemicals: This study considers the role of dopamine on motivated behaviour. However, other brain substances like serotonin or oxytocin influence social behaviour. Consequently, we propose the inclusion of these new chemicals to regulate Mini’s social behaviour, depending on the user responses and behaviour, and evaluate how people perceive these changes. Moreover, investigating the interactions among neurotransmitters and their impact on dopamine secretion is essential for improved model implementation.

## 7. Conclusions

This paper presents a bio-inspired decision-making model for autonomous robots focusing on dopamine’s role in action selection and reward seeking. We trained the model in simulation and later integrated it into the Mini social robot to obtain an autonomous agent able to regulate its internal biological processes and select the best action in each situation, adapting and learning from these experiences.

We designed a model that uses neuroscience and psychological studies to emulate human motivation and obtain autonomous agents that behave based on stimuli perception and an internal biological model that evolves with time. The model integrates and updates state-of-the-art dopamine models [6,7,24] to produce a biologically inspired motivational system. This system is based on dopamine mechanisms and includes adapting to dynamic situations, learning from both new and past experiences through a reinforcement learning approach, and anticipating possible future events previously experienced by the agent.

We designed five scenarios to test the model performance, including a real scenario for our robot to learn to behave and maintain optimal well-being. The first scenario shows a learning case where Mini learns to anticipate future rewards with experience. This case is critical for autonomous robots to avoid unexpected situations or execute action before an event occurs. The second scenario shows how the system deals with dynamic stimuli and rewards, learning how to generate appropriate dopamine levels in dynamic scenarios. The third case shows how dopamine adapts when the user provides negative feedback, driving the robot to avoid such negative situations. Finally, we designed a more complex environment with four biologically inspired processes and Mini has to correctly reduce the deficits appearing in them by selecting the most appropriate action.

The results show that this model has positive outcomes for real robots that interact and assist people in different scenarios. This setup demonstrates that the proposed method can be extended or modified to drive the behaviour of autonomous robots in other applications, such as mobile exploratory robotics, where motivation and curiosity are important factors, or in personalised learning robots, where dopamine-like learning enables a tailored user experience.

## Figures and Tables

**Table 1:** Summary of pros, challenges, and our improvement to the related work presented in this paper.

Reference	Pros	Challenges to Be Addressed	Improvements by Our Approach
Cañamero et al. [27]	Pioneered emotion-driven decision-making, foundational for animal- and human-inspired robotic processes.	Limited scalability to more complex robotic systems.	A more complex dopamine model improves anticipation and learning, allowing better decision-making in dynamic environments.
Gadanho [28]	Introduced reinforcement learning and cognitive elements, enabling agent evolution and adaptation.	Integration of emotion and cognition may introduce computational complexity. The learning method limits the addition of new processes.	A bio-inspired model facilitates the introduction of new behaviours by providing a modular architecture.
Lisetti et al. [30]	Enhanced social skills through emotional expression, improving human–robot interaction.	Limited expressiveness and understanding of diverse human emotions.	Decision-making is based on biological processes and dopamine dynamics that consider the effect of stimuli on anticipating future rewards.
Hiolle et al. [33]	Established an arousal-based model for decision-making, contributing to adaptive robotic behaviour.	Challenges in precisely mapping emotional states to arousal levels.	The biological model enables a more diverse behaviour control based on dynamic processes that evolve with time.
O’Brien and Arkin [35]	Modelled circadian rhythms, aligning robot behaviour with daily human needs.	Implementation challenges in accurately capturing and mimicking circadian patterns.	Including a dopamine model with learning improves the adaptation to dynamic environments and reacting to dynamic stimuli.
Hong et al. [37]	Developed a multimodal decision-making model, improving engagement through user-emotional state estimation.	Ensuring accurate estimation of user emotions and effective actuation based on these estimations.	The bio-inspired dopamine model allows the robot to make decisions based on the robot’s internal processes and ambient stimuli, not only considering user emotions.
Maroto-Gómez et al. [38]	Established a biologically-influenced decision-making system, continuously updated based on stimuli.	Challenges in precisely mimicking and adapting to biological factors in decision-making.	Including the dopamine model with learning allows the robot to anticipate future rewards and adapt to dynamic environments.

**Table 2:** Glossary of technical terms used by the model.

Term	Definition
Adaptation	The process by which an organism or system adjusts to changes in its environment or conditions to maintain functionality.
Anticipation	The ability to predict or expect a future event or outcome, often influencing behaviour or decision-making.
Biological process	A series of events or actions that occur in living organisms to maintain life, such as digestion, growth, or cellular repair.
The cerebral cortex	The outer layer of the brain that is responsible for complex functions like thought, perception, memory, and decision-making.
Deficit	A lack or shortage of something, often referring to a shortfall in a specific function, resource, or ability.
Dopamine	A neurotransmitter in the brain that plays a key role in motivation, reward, and learning processes.
Eligibility traces	A method in reinforcement learning that helps associate past actions with future rewards, improving learning efficiency.
Expected reward	The predicted value or benefit that an individual anticipates receiving due to a specific action or decision.
Gain matrix	In control theory, a matrix that is used to adjust the strength of control signals, optimising system performance.
Homeostasis	The process by which living organisms regulate internal conditions, such as temperature or pH, to maintain a stable, healthy state.
Motivation	The drive or desire to achieve a goal is often influenced by rewards, needs, or expectations.
Reinforcement learning	A type of machine learning where an agent learns to make decisions by receiving rewards or punishments for its actions.
Reward	A positive outcome or benefit from performing a particular action often reinforces that behaviour.
Striatum	A brain region is involved in planning and executing movements, processing rewards, and forming habits.
Ventral tegmental area	A part of the brain that plays a crucial role in the reward system, releasing dopamine in response to rewarding stimuli.
Well-being	A state of being comfortable, healthy, and happy, often encompassing both physical and mental health.

**Table 3:** Relationship between the internal processes, their deficits, variation rates, stimuli, and actions to maintain an optimal internal state regulating decision-making and action selection.

Internal Process	Deficit	Variation Rate	Range	Stimulus	Action	Action Effect
Sleep	Fatigue	+0.2	0 to 100	Lights off	Sleep	−8 fatigue deficit
Feeding	Hunger	−0.5	100 to 0	Broccoli	Eat	−10 hunger deficit
Moisturising	Thirst	−0.5	100 to 0	Water	Drink	−10 thirst deficit
Entertainment	Boredom	−0.3	100 to 0	User	Play	−6 on boredom deficit

**Table 4:** Model parameters used to dynamically generate anticipatory dopamine levels depending on the stimuli the robot perceives and the effects of its actions.

Variable	Value	Role
dt	1	Sampling period
Microt	20	Duration of each microstate
$\alpha_{v}$	0.03	Learning factor
λ	0.75	Eligibility trace retention
$\tau_{\delta }$	2	Time constant of dopamine secretion
τ	20	Time constant of reward
*s*	0.1	Pleasure scale factor
*k*	0.05	Pleasure decay factor
η	0.995	Discount factor to reduce reward dispersion in time

## Data Availability

The original contributions presented in the study are included in the article, further inquiries can be directed to the corresponding authors.
